# Cost-Utility Analysis of Community Case Management for Malaria Control in Burundi

**DOI:** 10.34172/ijhpm.2022.6290

**Published:** 2022-05-23

**Authors:** Nina Hezagira, Sitaporn Youngkong, Arthorn Riewpaiboon

**Affiliations:** ^1^Social, Economic and Administrative Pharmacy Program, Faculty of Pharmacy, Mahidol University, Bangkok, Thailand.; ^2^Division of Social and Administrative Pharmacy, Department of Pharmacy, Faculty of Pharmacy, Mahidol University, Bangkok, Thailand.

**Keywords:** Cost-Utility Analysis (CUA), Community Case Management (CCM), Malaria Control, Burundi

## Abstract

**Background:** The community case management (CCM) program for malaria control is a community-based strategy implemented to regulate malaria in children in Burundi. This study compared the cost and utility of implementing the CCM program combined with health facility management (HFM) versus HFM alone for malaria control in children under five in Burundi.

**Methods:** This study constructed a five-year Markov model with one-week cycles to estimate cost-utility and budget impact analysis (BIA). The model defined 10 health states, simulating the progression of the disease and the risk of recurrent malaria in children under five years of age. Cost data were empirically collected and presented for 2019. Incremental cost per disability-adjusted life year (DALY) averted, and a five-year budget was estimated. One-way and probabilistic sensitivity analyses (PSAs) were then performed.

**Results:** From provider and societal perspectives, combining the CCM program with HFM for malaria control in Burundi was more cost-effective than implementing HFM alone. The addition of CCM, using artesunate amodiaquine (ASAQ) as the first-line treatment, increased by US$1.70, and US$ 1.67 per DALY averted from the provider and societal perspectives, respectively. Using Artemether Lumefantrine (AL) as the first-line treatment, adding the CCM program to HFM increased by US$ 1.92, and US$ 1.87 per DALY averted from the provider and societal perspectives. At a willingness-to-pay of one GDP/capita, the CCM program remained a 100% chance of being cost-effective. In addition, implementing the program for five years requires a budget of US$ 15 800 486–19 765 117.

**Conclusion:** Implementing the CCM program and HFM is value for money for malaria control in Burundi. The findings can support decision-makers in Burundi in deciding on resource allocation, especially during the program’s scale up.

## Background

**Key Messages**

** Implications for policy makers**

- Implementation of the community case management (CCM) program together with the health facility management (HFM) for malaria control compared to the implementation of HFM alone in children aged under five years in Burundi was cost-effective with an incremental cost that varies between US$ 1.66 to US$1.92 per outcome in term of disability-adjusted life year (DALY) averted.
- To scale up the CCM program in Burundi and implement the program for five years requires a budget of US$ 15 800 486–19 765 117.
- The results can assist policy-makers in scaling up management for malaria control for children under five in Burundi.

** Implications for the public**

The community case management (CCM) program is essential for managing malaria control in endemic malaria areas, especially for children under five. Implementing the CCM program and health facility management (HFM) for malaria control in children under five in Burundi required less than US$ 2 to prevent life-year loss compared to the conventional program (HFM alone). This may lead to the policy decision on scaling up and then increasing the service accessibility of Burundi’s CCM program, which has only been implemented in a few provinces.

In sub-Saharan Africa, malaria is the leading cause of morbidity and mortality, especially in children under five. According to the Demographic and Health Survey performed in 2017, malaria’s prevalence in children under five in Burundi has increased from 17% in 2012 to 27% in 2017.^1^ Moreover, malaria accounted for 69% of all deaths in children aged under five in Burundi.^2^ In 2018, the country recorded 5 million cases and 3279 deaths due to malaria, and reached the epidemic threshold in 2019.^3,4^ Children were the most affected demographic.

 Burundi is a low-income country in East Africa; as of 2020, it had a per capita gross domestic product (GDP) of US$ 280^5^ and a population of 11.5 million.^6^ Prompt diagnosis and treatment with effective antimalarial medicines are essential for reducing the burden of malaria. In Burundi, since 2006, healthcare services for children under five have been free of charge at all public health facilities; however, in many low- and middle-income countries, including Burundi, timely access to healthcare is limited by financial constraints, geographical inaccessibility (90% of the Burundian population lives in rural areas where traditional houses are scattered between the hills and surrounded by crops), and lack of awareness about malaria complications.^7^ Moreover, the Burundian health system faces many challenges in improving healthcare accessibility, such as insufficient and poorly trained staff and frequent shortages of essential medicines.^8^ Hence, to overcome this problem, Burundi implemented a community case management (CCM) program for malaria control, as recommended by the World Health Organization (WHO).^9^

 The CCM program diagnoses and treats malaria in children aged under five years, near or at home, within 24 hours from the onset of fever. The service is provided by village health volunteers (VHVs), who work as community health workers. The VHVs do not have medical backgrounds and are trained to diagnose (using malaria rapid diagnostic test [RDT]) and treat uncomplicated malaria (using oral antimalarial medicines) in children.^10^ The program was initiated in 2013 as a pilot project and has been implemented along with standard health facility management (HFM) (the conventional approach for malaria control in Burundi). A 2014 study to evaluate the CCM pilot project found that the program effectively treated uncomplicated malaria and indirectly prevented severe malaria and death.^11^ The program is currently supported financially by non-government organizations, such as the United States Agency for International Development and the United Nations Children’s Fund; it has only been implemented in half of Burundi’s health districts.^11^ Moreover, with the march toward universal health coverage, the government is under pressure to expand or scale essential health services to meet the population’s needs. Therefore, the information on cost, cost-utility, and budget impact analysis (BIA) should be one of the criteria in choosing the efficient intervention to scale up; however, no studies have analyzed the cost-utility or the budget impact from the CCM program for malaria control in Burundi. Hence, this study conducted a cost-utility and BIA of the CCM program in combination with the HFM, compared to only implementing the HFM for malaria control, to support decision-makers in taking action in malaria management.

## Methods

###  Decision Model and its Description 

**Figure 1:**
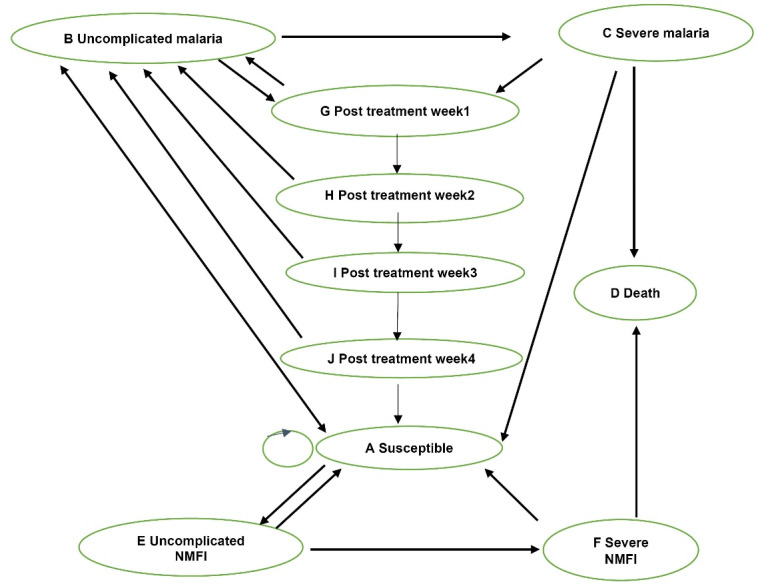
Markov Model. Abbreviation: NMFI, non-malaria febrile illness.

 This study conducted a cost-utility analysis using a Markov model for healthcare providers and societal perspectives (Figure 1).^12^ The model was adjusted from the previous economic evaluation studies to compare the CCM program implementation with HFM, versus the HFM alone, for malaria control as a conventional method in children under five in Burundi.^13,14^ The first-line antimalarial drug used at health facilities and the CCM program was artesunate amodiaquine (ASAQ),^3^ and the Burundian policy plan soon switched to artemether lumefantrine (AL). Therefore, this study analyzed the two scenarios of using different first-line treatments, that is, ASAQ and AL. RDT was solely used in the CCM program for malaria diagnostic tests; RDT and microscopic tests were used at health facilities in the proportion of 6.5:3.5.^15^

 The time horizon was five years, with one-week cycles, based on the assumption that the children do not benefit from the CCM program after reaching five years of age. The model starts with children aged 0 months in a healthy state. Possible events were modeled, including susceptible, uncomplicated malaria, severe malaria, uncomplicated non-malaria febrile illnesses (NMFIs), severe NMFI, and death; this model assumed that death could only occur due to severe malaria or severe NMFI. The model also included post-treatment states during weeks 1–4 in which a patient was at risk for recurrent malaria due to recrudescence or new infection.

 The model compared the costs and health outcomes (ie, disability-adjusted life years, DALYs) for children under five who benefit from each alternative. In combining the CCM program with the HFM arm, all the children started in a susceptible state. Children acquired a malaria diagnostic test depending on the incidence of fever, with all positive cases receiving antimalarial treatments. After malaria treatment in true positive cases, recurrent malaria might occur from weeks 1 to 4, depending on the antimalarial drugs used. For model simplicity, we assumed that recurrent malaria during these 4 weeks was appropriately treated. False-positive cases had other NMFI; however, among the NMFI, only the proportion caused by bacteria might progress to severe illness. The remaining viral infections were assumed to recover with or without any treatment. Among the negative cases, those involved in the CCM program were referred to a health facility. In this case, we assumed adherence to the negative test results and that all children received antibiotics (ie, amoxicillin, the most used antibiotic in Burundi).^16^ Children who did not access any health facility were assumed to go untreated and faced higher probabilities of developing severe illness. All the severe cases were admitted to in-patient care in a hospital because the program was designed to treat uncomplicated malaria.^13,17^ Children were assumed to stay in uncomplicated malaria or uncomplicated NMFI for one cycle.^18^ Inappropriately treated or untreated children in any state had higher probabilities of developing severity illness and dying.

 For the conventional arm (HFM alone), only febrile children who could access health care facilities were appropriately treated. The remaining children were assumed to go untreated and face a higher probability of developing severe malaria and dying. Concerning children who could access a health facility, the outcomes depended on the accuracy of diagnostic tests used (ie, RDT and microscopy), the incidence of fever, and the proportion of fever attributable to malaria. Similar to the other arm, true positive cases received antimalarial medicine.

###  Transitional Probabilities

 The transitional probabilities between different health states were derived from the literature, as shown in Table 1. We estimated the proportion of febrile children with access to a health facility and the weekly frequency of fever depending on the child’s age from the 2017 demographic health survey.^1^ The proportion of fever from malaria in Burundi was found in a study conducted in many African countries.^19^ The probability of appropriately treated uncomplicated malaria progressing to severe was derived from another modeling study on artemisinin resistance.^20^ Data of a large trial in many African countries on the effectiveness of artesunate versus quinine in treating severe malaria was used to estimate the probability of death from appropriately treated severe malaria.^21^ The probability of inappropriately treated or untreated uncomplicated malaria progressing to severe and the probability of dying from inappropriately treated or untreated severe malaria were derived from a published literature and Delphi survey.^22^

**Table 1:** List of All Parameters Used in the Model

**Parameters**	**Distribution**	**Mean**	**SE**	**Reference**
Weekly fever incidence				
Less than 12 months	Beta	0.188	0.012	^1^
Between 12–23 months	Beta	0.235	0.015	
Between 24–35 months	Beta	0.207	0.082	
Between 36–47 months	Beta	0.191	0.012	
Between 48–59 months	Beta	0.166	0.012	
Proportion of fever attributable to malaria	Beta	0.260	0.017	^19^
Probability that appropriately treated uncomplicated malaria progress to severe	Beta	0.020	0.011	^20^
Case fatality rate from appropriately treated severe malaria	Beta	0.085	0.005	^21^
Probability that inappropriately treated or untreated uncomplicated malaria progress to severe	Beta	0.075	0.007	^22^
Case fatality rate of inappropriately treated or severe untreated malaria	Beta	0.600	0.087	
Proportion of bacterial infection among NMFI	Beta	0.100	0.006	^23^
Probability that appropriately treated uncomplicated bacterial infection progress to severe	Beta	0.010	0.003	^24^
Case fatality rate from appropriately treated severe NMFI	Beta	0.100	0.017	^24^
Probability that inappropriately treated or untreated uncomplicated bacterial infection progress to severe	Beta	0.200	0.059	^22^
Case fatality rate of inappropriately treated or untreated severe NMFI	Beta	0.400	0.036	
Proportion of febrile children that have access to a health facility	Beta	0.58	0.083	^1^
Sensitivity of RDT	Beta	0.95	0.050	^26^
Specificity of RDT test	Beta	0.86	0.030	
Sensitivity of microscopic test	Beta	0.82	0.010	^27^
Specificity of microscopic test	Beta	0.85	0.040	
Proportion of RDTs used at Health facility	Beta	0.65	0.100	^15^
ASAQ				
Probability of recurrent malaria after week 1	Beta	0.040	0.006	^25^
Probability of recurrent malaria after week 2	Beta	0.019	0.004	
Probability of recurrent malaria after week 3	Beta	0.020	0.004	
Probability of recurrent malaria after week 4	Beta	0.099	0.009	
AL				
Probability of recurrent malaria after week 1	Beta	0.0220	0.0042	^25^
Probability of recurrent malaria after week 2	Beta	0.0117	0.0310	
Probability of recurrent malaria after week 3	Beta	0.0228	0.0440	
Probability of recurrent malaria after week 4	Beta	0.1347	0.0108	
Costs				
Cost of OPD visit (includes cost of routine services, cost of other drugs used [analgesic and antipyretic], and cost of other lab tests [if any]).	Gamma	2.180	3.600	Primary data
ASAQ cost	Gamma	0.345	0.077	
AL cost	Gamma	0.594	0.110	
Antibiotic cost	Gamma	0.705	0.202	
RDT cost in a health facility	Gamma	2.000	0.590	
Microscopy test in a health facility	Gamma	1.060	0.200	
Cost of fever diagnosis in the CCM program	Gamma	1.900	0.300	^11^
Cost of malaria treatment with ASAQ in CCM program	Gamma	1.345	0.250	^11^
Cost of malaria treatment with AL in CCM program	Gamma	1.594	0.250	^11^
Cost of Severe Malaria treatment	Gamma	69.128	15.000	Primary data
Cost of Severe NMFI treatment	Gamma	109.805	15.000	^31^
DNMC and IDC for uncomplicated malaria/NMFI at a health facility	Gamma	3.850	1.200	Primary data
DNMC and IDC for uncomplicated malaria/NMFI at CCM	Gamma	2.720	2.300	
DNMC and IDC for severe malaria	Gamma	23.010	10.000	
DNMC and IDC for severe NMFI	Gamma	28.520	10.000	
Disability weight				
Disability weight for uncomplicated febrile infectious illness	Beta	0.0050	0.0020	^34^
Disability weight for severe febrile infectious illness	Beta	0.2100	0.0500	
Discount rate** = **0.03				^35^
Birth cohort **= **522 873 children				^36^
Population growth rate/year = 0.028				^36^
Life expectancy at birth** = **60 years of age				^33^

Abbreviations: SE, standard error; AL, artemether lumefantrine; ASAQ, artesunate amodiaquine; CCM, community case management; DNMC, direct non-medical cost; IDC, indirect cost; NMFI, non-malaria febrile illness; RDT, rapid diagnostic test.

 The probability of bacterial infections among health states was obtained from published literature.^22-25^ The probability of recurrent malaria during weeks 1 to 4 was calculated from a Kaplan Meier curve of a large randomized control trial conducted in four African countries.^25^ Recurrent malaria in our model was set for four weeks because after this period, recurrence was mainly due to transmission in the area and not related to the antimalarial medicines used. The specificity and sensitivity of RDT and microscopic tests were derived from published studies,^26,27^ and the probabilities of the test results were included in the model using the Bayesian approach.

###  Costs 

 Cost data from each health state of the model was collected prospectively from two health facilities in Burundi: the Hospital Prince Regent Charles, a tertiary hospital with 600 beds having both out-patient and in-patient departments; and the Centre de Medecine Communautaire de Buyenzi, a primary health center with an out-patient only department between November and December 2019. The sample included 85 children below five diagnosed with malaria who visited and received treatment (as out- or in-patients) at either health facility during the study period. The patients with severe chronic illnesses (eg, HIV/AIDS or malnutrition) and patients with incomplete medical records were excluded.

 The costs used in this study were calculated from the provider and societal perspectives. The societal perspective included direct medical, non-medical, and indirect costs, while the provider perspective considered only direct medical costs. The direct medical costs consisted of the cost of malaria treatment in each state for the intervention arm (CCM with HFM program) and the conventional arm (HFM only). The CCM program cost comprised the cost of providing the program estimated based on the extant literature,^11^ and malaria cost in each state. The cost of malaria included the cost of antimalarial drugs (ASAQ tablets or AL tablets), supervision and management, cost of other materials, and financial incentives per case treated. The cost of the HFM program included the cost of malaria only. The resource used to estimate the direct medical cost was collected from the medical records of children below five diagnosed as malaria patients. The unit costs of routine medical services were obtained from WHO-CHOICE.^28^ The unit costs of antimalarial drugs, antibiotics, and RDT were included separately in the model and derived from the WHO international drug price^29^; we added 10%, representing the shipment and delivery price. The unit costs of other drugs, medical suppliers, and laboratory tests were estimated from the price in the private market. The cost of in-patient care for severe bacterial illness (NMFI) was estimated based on a study conducted in Kenya.^30^ The data on direct non-medical costs (DNMCs), that are expenses for food and transportation, were collected by interviewing the children’s caregivers. For indirect costs, the caregivers’ time lost when caring for the sick child was collected the same way. The opportunity cost of the caregivers’ time loss was estimated using the human capital approach by multiplying the Burundian GDP/capita/day^5^ by the number of days lost. This analysis did not include the opportunity cost due to the patients’ time loss.^31^ All the costs were presented in the 2019 value of the US dollar, at the exchange rate of 1 US dollar = 1845.62 Burundi Franc (BIF),^32^ discounted at 3%.

###  Disability-Adjusted Life Years Estimates

 This study measured the DALYs averted as the health outcomes; DALYs combine years of life lost because of premature mortality with years of life lived with disability.^31^ We used the average life expectancy for children under five in Burundi from WHO health statistics.^33^ The disability weights for uncomplicated and severe cases were derived from the WHO global burden of diseases.^34^ The DALYs were discounted at 3%, and no age weighting was applied.^35^

###  Analysis

 We estimated the incremental cost-utility ratio (ICUR) in terms of incremental cost per DALY averted from the healthcare provider and societal perspectives.^31^ We used a cost-effectiveness threshold (willingness to pay) of 1 GDP/capita. Hence, the intervention with an ICUR of less than one Burundian GDP/capita in 2019 (US$ 280)^5^ was considered cost-effective. BIA was also conducted to estimate the incremental budget or cost of the CCM program from the perspective of healthcare provider. The model followed the birth cohorts from all five years and followed each cohort for their duration in the study’s time horizon. The annual birth cohort and growth rate were derived from the available literature.^36^

###  Sensitivity Analysis

 The robustness of the results was tested using one-way and probabilistic sensitivity analysis (PSA) to determine how the ICUR changed by varying each parameter in the model. All the parameters were tried individually by applying minimum and maximum values. The results of the parameters with significant variation were presented using tornado diagrams. The input parameters for PSA were used with Monte Carlo simulation with 10 000 iterations. The results were presented as a cost-effectiveness plane and cost-effectiveness acceptability curve.

## Results

###  Base Case Analysis

 Implementing the CCM program increased the total costs of the conventional approach (HFM); however, DALYs lost were much lower when combining the CCM program with the HFM, as shown in Table 2. From the provider perspective, the ICURs for implementing the CCM program combined with HFM were US$ 1.70 and US$ 1.92 per DALY averted when using ASAQ and AL as first-line treatments, respectively, and the ICURs from a societal perspective were US$ 1.67 and US$1.87 per DALY averted. Based on the cost-effectiveness threshold of 1 GDP per capita, implementing the CCM program and HFM was consistently more cost-effective than HFM alone.

**Table 2:** Results of Cost-Utility Analysis of the Base Case in 2019 (US$)

**Strategy**	**Total Costs **(**US$**)	**DALYs**	**Incremental Cost** (**US$**)	**Incremental** **DALY Averted**	**ICUR **(**Incremental Cost/DALY Averted**)
**Provider Perspective**					
ASAQ as the first-line treatment					
HFM alone	119.22	10.37	Reference	Reference	Reference
Combination of the CCM program and HFM	128.47	4.94	9.25	5.43	1.70
AL as the first-line treatment					
HFM alone	121.05	10.36	Reference	Reference	Reference
Combination of the CCM program and HFM	131.46	4.96	10.41	5.40	1.92
**Societal Perspective**					
ASAQ as the first-line treatment					
HFM alone	204.53	10.37	Reference	Reference	Reference
Combination of the CCM program and HFM	213.60	4.94	9.07	5.43	1.67
AL as the first-line treatment					
HFM alone	206.40	10.36	Reference	Reference	Reference
Combination of the CCM program and HFM	216.51	4.96	10.11	5.40	1.87

Abbreviations: AL, artemether lumefantrine; ASAQ, artesunate amodiaquine; CCM, community case management; IDC, indirect cost; NMFI, non-malaria febrile illness; RDT, rapid diagnostic test; HFM, Health facility management; ICUR, incremental cost-utility ratio; DALY, disability-adjusted life year.

###  Sensitivity Analysis 

**Figure 2:**
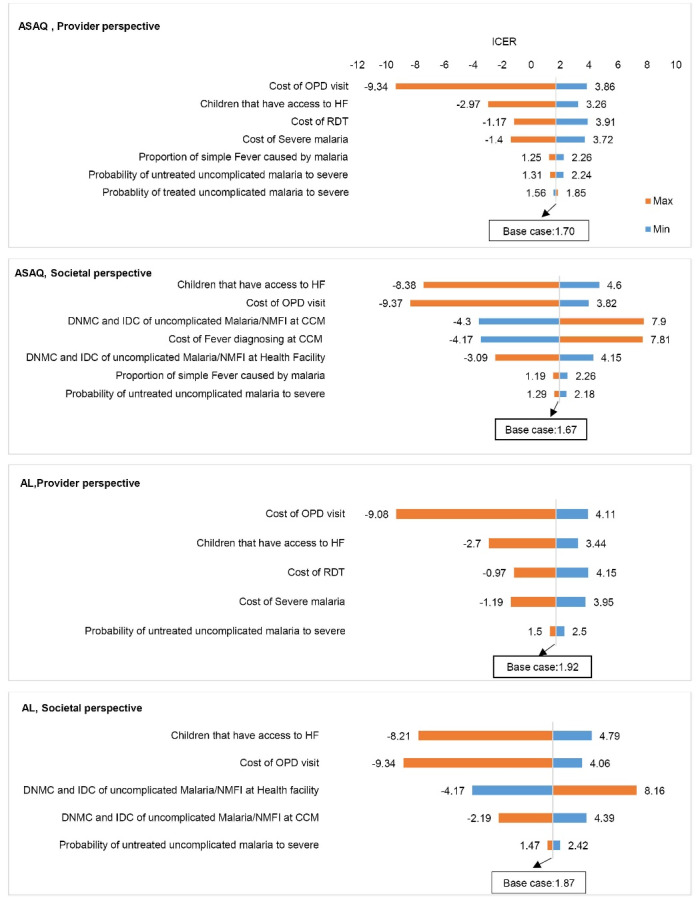
Results of One-Way Sensitivity Analysis. Abbreviations: CCM, community case management; HF, health facility; RDT, rapid diagnostic test; DNMC, Direct non-medical cost; IDC, indirect cost; ASAQ, artesunate amodiaquine; OPD, out-patient department; AL, artemether lumefantrine; NMFI, non-malaria febrile illness; ICER, incremental cost-effectiveness ratio.

 The results of the one-way sensitivity analysis indicate that the most influential parameter on ICUR was the probability of accessing a health facility, cost of out-patient department (OPD) visit, direct non-medical and indirect cost for uncomplicated fever at the CCM program and health facility, and cost of RDT; however, the program remained cost-effective in all variations. The results were the same in both perspectives (Figure 2).

**Figure 3:**
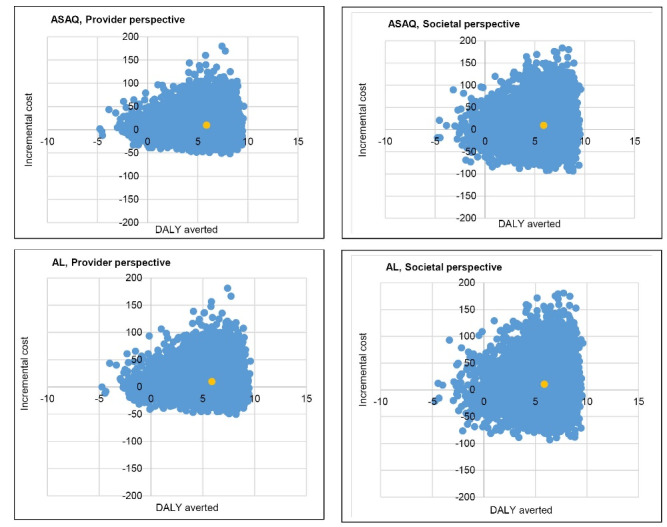
Cost-Effectiveness Plane. Abbreviations: AL, Artemether lumefantrine; ASAQ, artesunate amodiaquine; DALY, disability-adjusted life-year.

 The cost-effectiveness planes analyzed from PSA indicate that the CCM program tended to be cost- effective (Figure 3).

**Figure 4:**
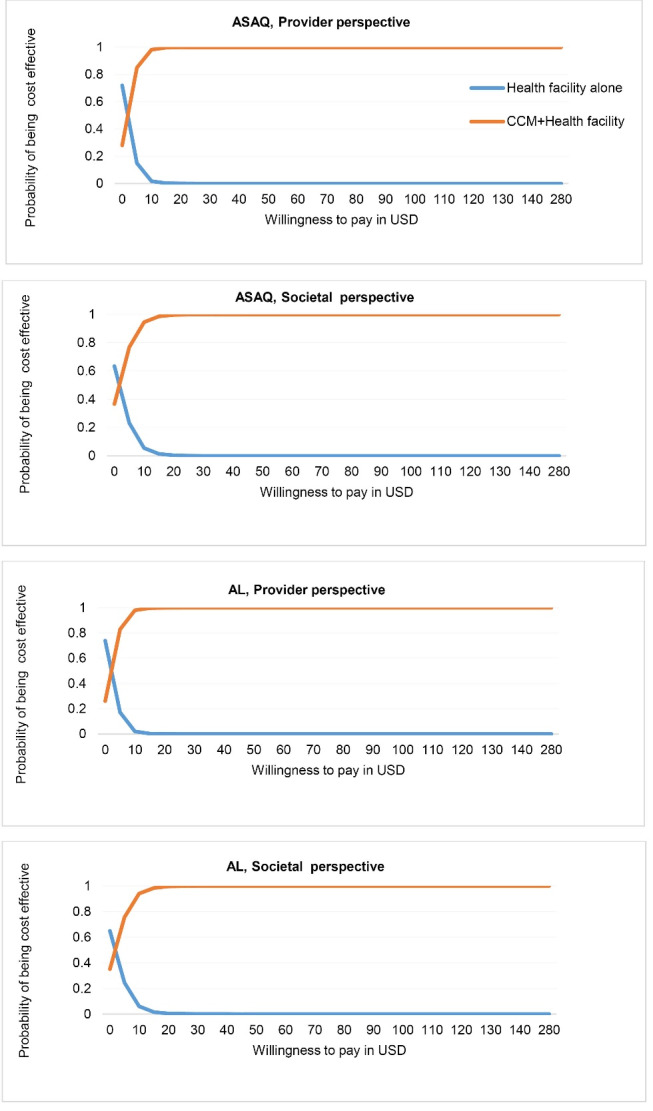
Cost-Effectiveness Acceptability Curve. Abbreviations: AL, artemether lumefantrine; ASAQ, artesunate amodiaquine; CCM, community case management.

 From the cost-effectiveness acceptability curves (Figure 4), at a willingness to pay of 1 GDP/capita (US$ 280), the combination of the CCM program and HFM has a 100% chance of being cost-effective for both first-line treatments and perspectives.

###  Budget Impact Analysis

 Using ASAQ as the first-line treatment, the implementation of the CCM program requires an incremental budget of US$ 543 071, US$ 1 786 993, US$ 3 263 218, US$ 4 899 276, and US$ 5 307 926 for the first to the fifth year, respectively, for a total of US$ 15 800 486. Conversely, using AL as first-line treatment, the implementation of the CCM program requires an incremental budget of US$ 654 232, US$ 1 593 008, US$ 2 878 427, US$ 4 427 864, and US$ 10 211 584 for the first to the fifth year, respectively, for a total of US$ 19 765 117.

## Discussion

 This study is the first in Burundi to determine the cost-utility of the CCM program for malaria control. We used a Markov model to mimic malaria disease progression and compared the cost and health outcomes of different malaria control strategies. This study’s results can be compared with a similar study in Uganda.^13^ The study indicated that in high transmission areas, when the probability of accessing health care is greater than 50%, the combination of the CCM program with HFM using any first-line treatment, that is, AL or ASAQ, was more cost-effective than HFM alone; however, there is a difference in the design of the CCM programs implemented in Uganda and Burundi. The program in Uganda presumptively distributed antimalarial drugs to all febrile children, whereas the program in Burundi diagnosed before treating a malaria case. This may support the utilization of the CCM program is likely cost-effective in controlling malaria in the high transmission areas, even though they are implemented differently in practice. In Uganda, the program was cost-saving when the probability of accessing a health facility is 50% with an ICUR equal to US$ –63 in high transmission intensity and US$ −28 in medium transmission intensity (in 2010 value, GDP/capita of Uganda = US$ 794.34). Moreover, other economic evaluation studies in Ghana^37,38^ and Zambia^39^ on the CCM program for malaria control also presented that implementing the CCM program for malaria control tended to be cost-effective with different estimates. In Ghana, the cost per DALY averted was US$ 90.25 in 2012 (GDP/capita = US$ 2202.12), while in Zambia, the cost per case correctly diagnosed and treated was US$ 4.22 in 2011 (GDP/capita = US$ 1305.06). Different analytical methods and perspectives can explain these wide differences in the cost-utility analysis. Moreover, the CCM program established in those countries had different designs. The program in Uganda treated all fever cases presumptively, and VHVs were unpaid volunteers. In contrast, the CCM programs in Zambia and Ghana were designed to diagnose before treating, and VHVs received salaries or incentives.

 This study showed that using either ASAQ or AL as the first-line treatment tended to be cost-effective from both provider and societal perspectives. Using ASAQ as the first-line treatment would cost less than AL, but AL averted more DALY than ASAQ. Currently, the first-line medicine for uncomplicated malaria is ASAQ; however, there has been a problem with amodiaquine resistance in East Africa,^40^ and, therefore, AL appears to be a good substitute.

 The findings from sensitivity analysis suggest that the cost-effectiveness results were mainly affected by the probability of accessing healthcare services and the cost of OPD visits. As the cost of OPD visits increases, implementing the CCM program becomes more cost-saving because it can offer similar services at a lower cost. Similarly, implementing the CCM program becomes more cost-saving as the probability of accessing healthcare services increases, implying that this probability is very low in rural provinces. The CCM program would be more costly and effective but would remain cost-effective. It would have a high impact in terms of health benefits (as more children who were left untreated in a conventional method would be treated), but health care costs would rise due to expanding the service coverage. In areas where the probability of accessing healthcare is high, the program would be cost-saving. There was not much change in terms of health benefits, but healthcare costs would decrease because the cost of treatment through the CCM program is less than health facility treatment costs.

 There is a plan to increase the activity of the CCM program with the addition of two more diseases: diarrhea and pneumonia (called integrated CCM).^11^ This would further increase the cost-effectiveness of the malaria control program as the program’s cost per service would be decreased due to the economy of scope.

 This study used the conventional threshold of 1 GDP/capita, but new studies suggest using a threshold of no higher than half of GDP/capita in low-income countries.^41^ In all cases, our findings would remain cost effective; however, cost effective results do not always reflect the ability to afford the new intervention; therefore, we also conducted BIA. This study’s BIA results indicated a need for an incremental budget of US$ 15 800 486–19 765 117 to manage the birth cohorts from all five years under the CCM program. This budget is less than 1% of the Burundian GDP,^5^ but it may burden the government as the budget allocated to the Ministry of Health is already constrained.^42^

 This study makes several assumptions to simplify the model. The first simplification was to exclude the neurological sequelae caused by severe illnesses; this represents a small proportion and does not affect the model. Death from other causes was excluded, as it was assumed to be the same in both arms.^13^ Case of co-infection of malaria with another NMFI was also excluded. It was difficult to quantify and might make the model too complex; however, they are sometimes overlooked in practical diagnosis as most health professionals use malaria test results in decision making.^43^ For simplicity, some movements in the model were limited, like a child cannot pass directly from malaria to NMFI, or vice versa, without passing to the susceptible state.

 Most parameters were not available for Burundi, so we were forced to use parameters from other countries. We tried to choose countries similar to Burundi when borrowing parameters. We applied disability weight for uncomplicated and severe illnesses, including malaria, derived from the recent WHO Global burden of disease^34^; however, some parameters were derived from the Delphi survey or expert opinion, which is a limitation of those parameters. To overcome this, we conducted a probabilistic and one-way sensitivity analysis to consider the effect of plausible parameter variation on the results, which were not much affected by plausible variation in the parameter. Therefore, they can be generalized to other settings with almost identical malaria epidemiology and healthcare infrastructures.

## Conclusion

 Our findings show that implementing the CCM program for malaria control is cost effective in Burundi. Policy-makers have started implementing the program in Burundi without considering its investment worth; therefore, this study’s findings can support the policy-makers’ decisions on resource allocation, especially when considering further implementation and scaling up the program.

## Acknowledgments

 We thank the staff at the Hospital Prince Regent Charles, the Centre de Medecine Communautaire de Buyenzi and community health workers in Gitega Burundi, and the staff at the Ministry of Health for their support during cost data collection. We also thank the Thailand International Cooperation Agency and the Social, Economic, and Administrative Pharmacy postgraduate program, Faculty of Pharmacy, Mahidol University, Bangkok, Thailand, for funding this study.

## Ethical issues

 The Institutional Review Board of the Faculty of Dentistry/Faculty of Pharmacy, Mahidol University approved the study (COA.No.MU-DT/PY-IRB2019/071.1810).

## Competing interests

 Authors declare that they have no competing interests.

## Authors’ contributions

 NH and SY conceived and designed the work: NH conducted the analysis; NH and SY wrote the first draft of the manuscript with input from AR. All authors interpreted the data and approved the final version to be published.

## Funding

 This work was carried out as a part of study in Master of Science program in Social, Economic and Administrative Pharmacy (SEAP), Faculty of Pharmacy, Mahidol University, Bangkok, Thailand. The study received a research grant from the SEAP program and was also supported by the Thailand International Cooperation Agency (TICA).
